# Optimal LDL cholesterol levels in young and old patients with type 2 diabetes for secondary prevention of cardiovascular diseases are different

**DOI:** 10.1530/EC-23-0142

**Published:** 2023-09-27

**Authors:** Chaiho Jeong, Bongseong Kim, Jinyoung Kim, Hansang Baek, Mee Kyoung Kim, Tae-Seo Sohn, Ki-Hyun Baek, Ki-Ho Song, Hyun-Shik Son, Kyungdo Han, Hyuk-Sang Kwon

**Affiliations:** 1 Division of Endocrinology and Metabolism, Department of Internal Medicine, College of Medicine, The Catholic University of Korea, Seoul, Republic of Korea; 2 Department of Medical Statistics, Soongsil University of Korea, Seoul, Republic of Korea

**Keywords:** type 2 diabetes mellitus, LDL cholesterol, diabetes complications, cardiovascular diseases

## Abstract

**Objective:**

Real-world-based population data about the optimal low-density lipoprotein cholesterol (LDL-C) level for preventing cardiovascular disease in very high-risk populations is scarce.

**Methods:**

From 2009 to 2012, 26,922 people aged ≥ 40 years with type 2 diabetes mellitus (T2DM) who had a history of percutaneous coronary intervention (PCI) were analyzed. Data from the Korean National Health Insurance System were used. They were followed up to the date of a cardiovascular event or the time to death, or until December 31, 2018. Endpoints were recurrent PCI, newly stroke or heart failure, cardiovascular death, and all-cause death. Participants were divided into the following categories according to LDL-C level: <55 mg/dL, 55–69 mg/dL, 70–99 mg/dL, 100–129 mg/dL, 130–159 mg/dL, and ≥ 160 mg/dL.

**Results:**

Compared to LDL-C < 55 mg/dL, the hazard ratios (HR) for re-PCI and stroke increased linearly with increasing LDL-C level in the population < 65 years. However, in ≥ 65 years old, HRs for re-PCI and stroke in LDL-C = 55–69 mg/dL were 0.97 (95% CI: 0.85–1.11) and 0.96 (95% CI: 0.79–2.23), respectively. The optimal range with the lowest HR for heart failure and all-cause mortality were LDL-C = 70–99 mg/dL and LDL-C = 55–69 mg/dL, respectively, in all age groups (HR: 0.99, 95% CI: 0.91–1.08 and HR: 0.91, 95% CI: 0.81–1.01).

**Conclusion:**

LDL-C level below 55 mg/dL appears to be optimal in T2DM patients with established cardiovascular disease aged < 65 years, while an LDL-C level of 55–69 mg/dL may be optimal for preventing recurrent PCI and stroke in patients over 65 years old.

## Introduction

Cardiovascular disease (CVD) is one of the most significant causes of death globally. Despite its critical fatality rate, CVD can be prevented by taking necessary precautions (1). Several studies have indicated that a high level of low-density lipoprotein cholesterol (LDL-C) is strongly associated with CVD. Hundreds of studies with millions of participants have suggested that LDL-C is a strong predictor of atherosclerosis that eventually leads to atherosclerotic cardiovascular disease (ASCVD). Therefore, several guidelines underscore the importance of intensive LDL-C lowering for primary and secondary prevention of CVD.

It is well known that patients with established CVD have a high risk of subsequent CVD events, including myocardial infarction, stroke, and death (1, 2). A recent study has estimated that the 10-year risk of recurrent vascular events in patients with pre-experienced coronary artery disease is 14% (2). Thus, secondary prevention for this population is crucial for reducing recurrent cardiovascular events (CVE). Since the Scandinavian Simvastatin Survival Study showed that lowering LDL-C could significantly decrease both cardiovascular mortality and all-cause mortality, several studies including meta-analyses have demonstrated that the relative reduction in CVD risk is proportional to the absolute reduction of LDL-C (3, 4, 5). Moreover, recent research studies on PCSK-9 inhibitors have shown the concept of ‘the lower, the better’ without an apparent lower threshold for LDL-C level for secondary prevention (6, 7, 8).

Type 2 diabetes mellitus (T2DM) is an independent risk factor for ASCVD. T2DM patients are at a two-fold to four-fold increased risk of developing CVD (9). As a result, CVD is the most important cause of morbidity and mortality in individuals with T2DM. T2DM patients are likely to have multiple ASCVD risk factors (including dyslipidemia and hypertension), each of which increases the risk of both ASCVD and non-ASCVD. As a result, several recent guidelines including the American Diabetes Association ‘Standards of Care in Diabetes 2023’ recommends an LDL-C goal of less than 55 mg/dL in people with diabetes and established ASCVD (10, 11, 12, 13).

However, real-world data about the optimal LDL-C target level for preventing CVE in this very high-risk population is insufficient. In particular, the debate over the clinical benefits of LDL-C lowering in older patients with diabetes for secondary prevention remains. Therefore, we conducted a close investigation on the association between LDL-C and the onset of CVE as well as the mortality in T2DM patients with established CVD by utilizing and examining the data from the Korean National Health Insurance System. In addition to this, an analysis was performed by age group classification. This big data study is expected to support the recommended LDL-C level in this population.

## Materials and methods

### Data sources

The National Health Insurance Service (NHIS) in South Korea covers about 97% of the Korean population and provides health screening examinations called the National Health Screening Program for all enrollees aged 40 years and older. The NHIS includes an eligibility database (age, sex, socioeconomic variables, type of eligibility, and so on), a medical treatment database (based on medical bills claimed by medical service providers for medical expenses), a health examination database (general health examinations and questionnaires on lifestyle and behavior), a medical care institution database, and death information. Dates of death were obtained from the database prepared by Statistics Korea.

### Study population

**Figure 1:**
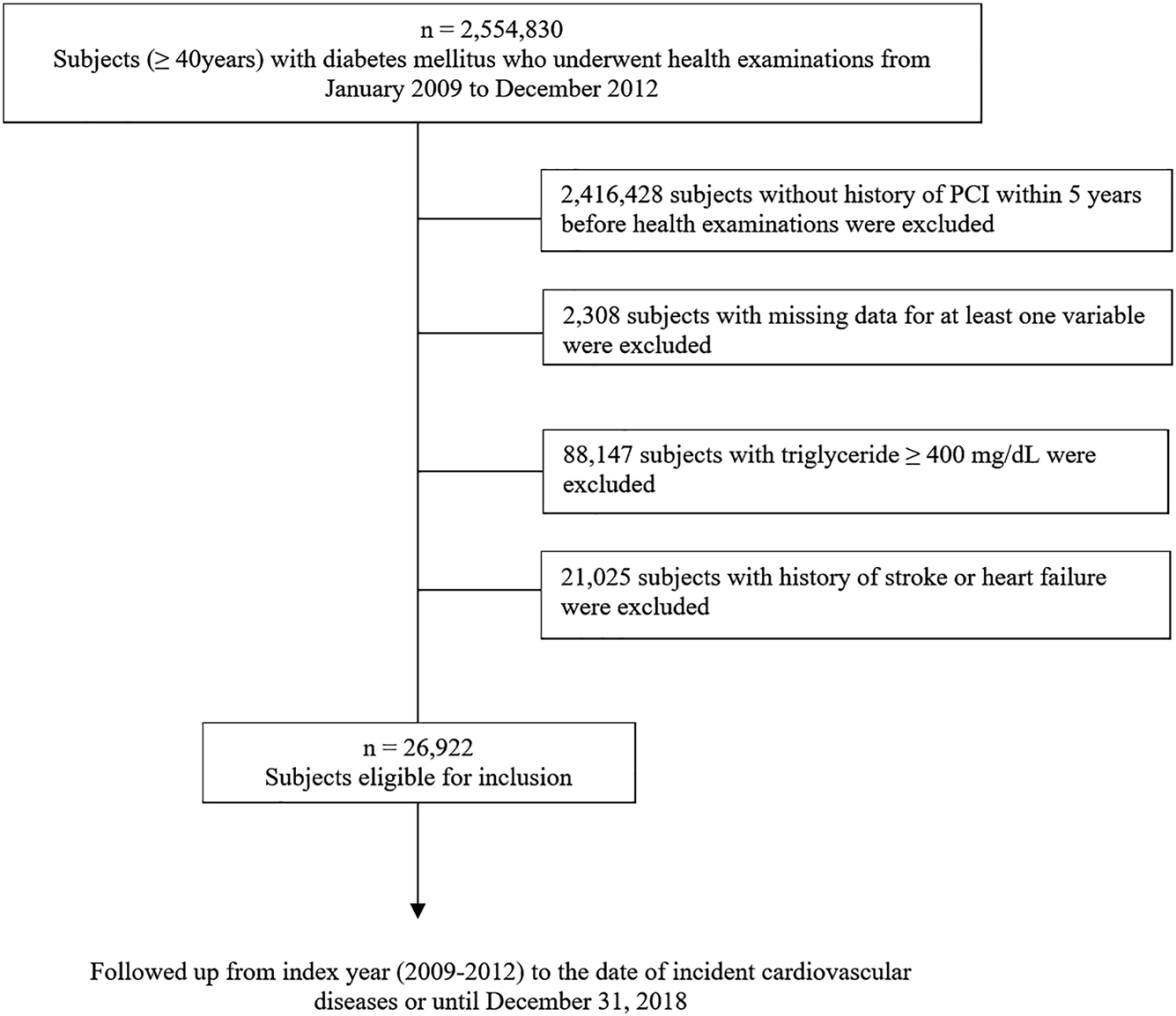
Flowchart showing the selection of the study population.

From January 1, 2009, to December 31, 2012, a total of 2,554,830 people aged ≥ 40 years with T2DM underwent a health examination. Patients who had at least one service claim with a diagnosis of T2DM based on ICD-10 (E11–E14) and patients prescribed at least one antidiabetic drug at any time over 1 year were classified as having T2DM. For example, among those who underwent a health examination in 2009, we selected participants who had at least one service claim with a diagnosis of T2DM based on ICD-10 (E11–14), and those who were prescribed at least one antidiabetic drug at any time in 2009. The same criteria were used for 2010, 2011, and 2012. Duplicate individuals who underwent multiple health examinations in consecutive years were excluded. The index year was 2009–2012. We used the same method described by Kim *et al.* (14). To include subjects with established CVD, those who did not have a history of percutaneous coronary intervention (PCI) within 5 years before health examination were excluded (n = 2,416,428). People with missing data for at least one variable were also excluded (*n* = 2308). Those with TG levels of > 400 mg/dL were excluded (*n* = 88,147). To avoid confounding by preexisting diseases and to minimize possible effects of reverse causality, we also excluded those with histories of heart failure (HF) or stroke (*n* = 21,025) as indicated by their medical treatment and health examination data before the index year. Ultimately, a total of 26,922 people were eligible for this study. The flowchart for the selection of the study population is described in Fig. 1. This study was approved by the Institutional Review Board of the Catholic University of Korea (No. UC22ZISI0112). Anonymized and deidentified information were used for analyses; therefore, informed consent was not obtained.

### Data collection

The covariates were based on data from the index year. They included age, sex, income level (low 25% or not), body mass index (BMI; kg/m^2^), smoking status (no, yes), alcohol consumption (no, yes), regular exercise, use of insulin (no, yes), duration of diabetes (year), and systolic/diastolic blood pressure (mmHg). The duration of diabetes was defined as the period time from January 1, 2002, to the time of the subject’s health examination, since the record from NHIS is only available from 2002. Regular exercise was defined as having 20 min or more of vigorous activity at least 3 days a week, or 30 min or more of moderate activity 5 or more days per week. Income level was dichotomized at the lowest 25%. We defined a statin user as a person who had a prescription of a statin within 1 year of health examination. Blood samples for measurements of serum glucose, creatinine, and lipid levels were drawn after an overnight fast. Blood samples for measurements of total cholesterol, high-density cholesterol (HDL-C), and triglyceride (TG) levels were obtained at the health examination after the participant had fasted for at least 8 h. LDL-C levels were calculated with the Friedewald formula: LDL-C = total cholesterol − HDL-C − (TG/5) (15).

### Study endpoints and follow-up

Endpoints of this study were as follows: event of recurrent PCI, newly diagnosed stroke or HF, cardiovascular death, and all-cause death. The detailed definitions of outcomes are described in Supplementary Table 1 (see section on supplementary materials given at the end of this article). The study population was followed up from baseline to the date of a CVE or the time of the participant’s disqualification from receiving health services due to death or emigration, or until the end of the study period (December 31, 2018).

### Statistical analysis

Baseline characteristics are presented as mean ± s.d., geometric mean (95% confidence interval (CI)), or *n* (%). Participants were divided into the following categories according to their LDL-C levels: < 55 mg/dL, 55–69 mg/dL, 70–99 mg/dL, 100–129 mg/dL, 130–159 mg/dL, and ≥ 160 mg/dL. The incidence rate (IR) of primary outcomes was calculated by dividing the number of incident cases by the total follow-up duration (person-years). The cumulative incidence of each CVE during follow-up according to LDL-C categories was assessed using Kaplan–Meier curves. The log-rank test was performed to evaluate differences among groups. Cox regression analyses were performed to estimate the risk of CVE and all-cause mortality for each LDL-C group using the group with LDL-C less than 55 mg/dL as the reference group. A multivariable-adjusted proportional hazards model was applied after it was adjusted for age, sex, BMI, smoking, alcohol consumption, regular exercise, household income, use of statins, fasting glucose levels, hypertension, use of insulin, and duration of diabetes. The potential effect modification by age was evaluated through stratified analysis and interaction testing using a likelihood ratio test. All statistical analyses were performed using SAS version 9.4 (SAS Institute Inc., Cary, NC, USA). A *P* < 0.05 was considered to indicate statistical significance.

## Results

### Baseline characteristics

Baseline characteristics of study subjects are presented in Table 1. Baseline characteristics according to gender are also shown in Supplementary Tables 2 and 3. For the cohort of 26,922 participants, the mean age was 63.0 ± 9.0 years and 19,425 (72.2%) participants were men. The mean LDL-C level was 81.0 ± 37.7 mg/dL. Patients in higher LDL-C categories were more likely to have higher fasting glucose levels. Conversely, patients with low LDL-C levels were more likely to have higher incomes, lower BMI, and engage in regular exercise. The baseline total cholesterol and triglyceride levels show significant differences according to LDL-C category.

**Table 1:** Baseline characteristics of subjects according to the low-density lipoprotein cholesterol (LDL-C) levels.

	Total participants (*N* = 26,922)	LDL-C < 55 (*N* = 5203)	LDL-C < 70 (*N* = 5894)	LDL-C 70–99 (*N* = 9696)	LDL-C 100–129 (*N* = 4150)	LDL-C 130–159 (*N* = 1361)	LDL-C **≥**160 (*N* = 618)	*P*
Baseline LDL-C (mg/dL)	81.0 ± 37.7	43.0 ± 9.7	62.2 ± 4.3	82.9 ± 8.4	112.0 ± 8.4	141.9 ± 8.4	206.6 ± 14.3	<0.0001
Age (years)	63.0 ± 9.0	62.7 ± 8.9	63.1 ± 8.9	63.0 ± 9.0	63.1 ± 9.2	63.3 ± 9.3	62.2 ± 9.9	0.015
Sex (male)	19,425 (72.2)	4090 (78.6)	4386 (74.4)	6885 (71.0)	2791 (67.3)	889 (65.3)	384 (62.1)	<0.0001
Body mass index (kg/m^2^)	25.1 ± 3.0	25.0 ± 2.9	25.1 ± 3.0	25.2 ± 3.0	25.2 ± 3.1	25.2 ± 3.2	25.2 ± 3.3	0.002
Fasting glucose (mg/dL)	135.7 ± 44.5	133.7 ± 42.8	133.4 ± 42.6	134.9 ± 42.9	138.8 ± 47.8	142.4 ± 49.3	151.3 ± 58.4	<0.0001
eGFR (mL/min/1.73 m^2^)	78.9 ± 34.7	80.1 ± 38.3	78.9 ± 31.1	78.9 ± 31.6	78.3 ± 40.6	77.4 ± 40.6	77.5 ± 26.9	0.056
Baseline TC (mg/dL)	155.5 ± 37.1	119.4 ± 17.8	135.2 ± 16.8	156.9 ± 18.0	188.7 ± 18.1	220.8 ± 19.6	266.4 ± 48.0	<0.0001
Baseline HDL-C (mg/dL)	47.4 ± 17.1	46.6 ± 15.5	46.4 ± 13.6	47.4 ± 14.4	48.2 ± 17.1	48.6 ± 26.9	54.1 ± 45.7	<0.0001
Baseline TG (mg/dL)	125.2 (124.4–125.9)	130.1 (128.2–132.0)	118.2 (116.7–119.7)	120.6 (119.5–121.7)	131.0 (129.2–132.8)	142.6 (139.3–146.0)	154.3 (149.1–159.7)	<0.0001
Hypertension	22,456 (83.4)	4333 (83.3)	4927 (83.6)	8150 (84.1)	3419 (82.4)	1125 (82.7)	502 (81.2)	0.111
Current smoker	4955 (18.4)	1038 (20.0)	1033 (17.5)	1690 (17.4)	752 (18.1)	302 (22.2)	140 (22.7)	<0.0001
Alcohol drinking	8275 (30.7)	1783 (34.3)	1881 (31.9)	2882 (29.7)	1191 (28.7)	376 (27.6)	162 (26.2)	<0.0001
Regular exercise	6544 (24.3)	1317 (25.3)	1504 (25.5)	2378 (24.5)	940 (22.7)	283 (20.8)	122 (19.7)	<0.0001
Income (lower 25%)	5187 (19.3)	986 (19.0)	1122 (19.0)	1832 (18.9)	819 (19.7)	278 (20.4)	150 (24.3)	0.022
On insulin treatment	6022 (22.4)	1260 (24.2)	1316 (22.3)	2126 (21.9)	872 (21.0)	303 (22.3)	145 (23.5)	0.006
On statin treatment	2339 (86.9)	4971 (95.5)	5578 (94.6)	8650 (89.2)	3022 (72.8)	799 (58.7)	377 (61.0)	<0.0001
Duration of diabetes (years)	4.8 ± 3.5	5.2 ± 3.4	4.9 ± 3.4	4.7 ± 3.5	4.7 ± 3.5	4.6 ± 3.4	4.2 ± 3.4	<0.0001

Data are expressed as the mean ± s.d., median (25–75%), or *n* (%).

eGFR, estimated glomerular filtration rate; HDL, high-density lipoprotein; LDL, low-density lipoprotein; TC, total cholesterol; TG, triglyceride.

### LDL-C level and risk of CVE

**Figure 2:**
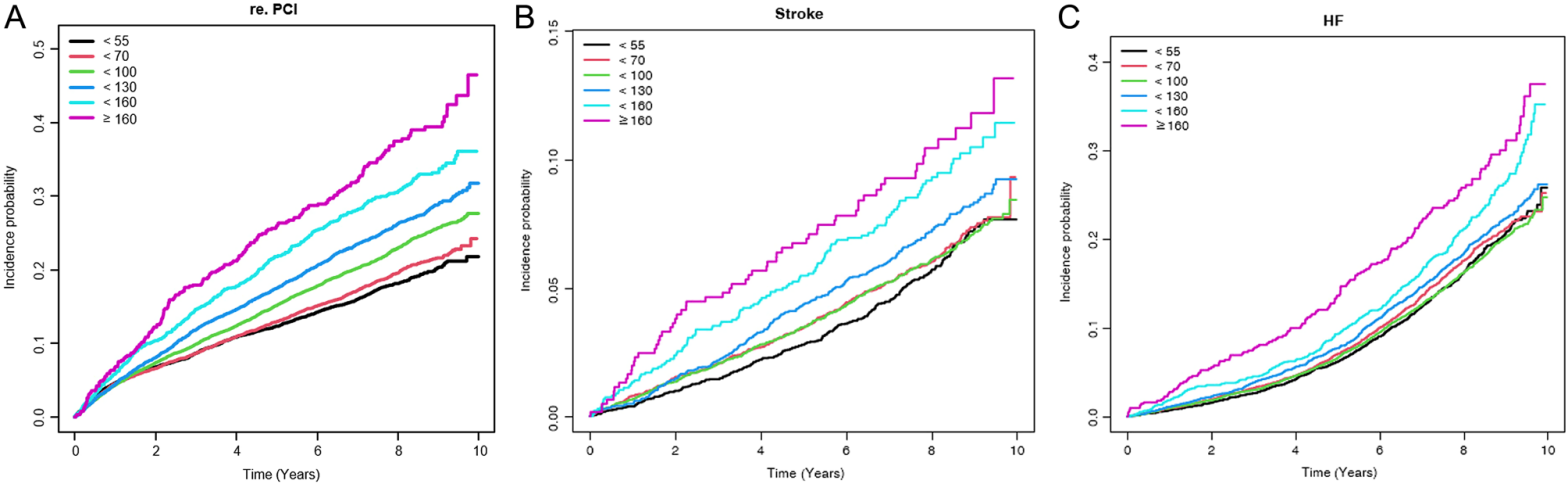
Cumulative incidence of (A) recurrent percutaneous coronary intervention, (B) stroke, and (C) heart failure during follow-up. The log-rank test was used to evaluate differences between groups. All results in the figure showed a *P* < 0.001.

Table 2 shows the incidence of re-PCI, stroke, HF, cardiovascular death, and all-cause mortality according to LDL-C level. The mean follow-up time was 7.6 ± 1.8 years for the total population. The final Model 3 was adjusted for age, sex, BMI, smoking, alcohol drinking, exercise, income status, hypertension, estimated glomerular filtration rate, fasting glucose levels, use of insulin, duration of diabetes, and use of statin. All models showed similar trends toward LDL–C levels. Using LDL-C level < 55 mg/dL as a reference, multivariable-adjusted hazard ratios (HR) and IR for re-PCI, stroke, and cardiovascular death increased linearly according to LDL-C level. HRs for re-PCI, stroke, and cardiovascular death in those with LDL-C ≥ 160 mg/dL were 2.12 (95% CI: 1.82–2.47), 1.68 (95% CI: 1.26–2.23), and 1.71 (95% CI: 1.17–2.48), respectively, in Model 3. However, the optimal LDL-C ranges with the lowest HR for HF and all-cause mortality were 70–99 mg/dL and 55–69 mg/dL, respectively (HR: 0.99, 95% CI: 0.91–1.08 and HR: 0.91, 95% CI: 0.81–1.01, respectively). In those with LDL-C ≥ 160 mg/dL, HRs for HF and all-cause mortality were 1.60 (95% CI 1.34–1.91), and 1.38 (95% CI 1.13–1.68) respectively. Figure 2 shows the cumulative incidence of re-PCI, stroke, and HF during follow-up. All the results in the figure showed a *P* < 0.001 (log-rank test). The incidence of outcomes according to gender is described in Supplementary Table 4. The results were similar to those for the general population.

**Table 2:** Risk of re-PCI, stroke, heart failure, cardiovascular death, and all-cause of mortality in patients with type 2 diabetes mellitus according to low-density lipoprotein cholesterol (LDL-C) category.

	LDL-C	*N*	Event	IR	Model 1	Model 2	Model 3
					HR (95% CI)^a^	HR (95% CI)^b^	HR (95% CI)^c^
**Re-PCI**							
	<55	5203	897	25.56	1 (ref.)	1 (ref.)	1 (ref.)
	55–69	5894	1102	27.43	1.08 (0.99–1.18)	1.10 (1.01–1.20)	1.10 (1.00–1.20)
	70–99	9696	2134	32.88	1.30 (1.20–1.40)	1.32 (1.22–1.43)	1.30 (1.20–1.40)
	100–129	4150	1041	38.23	1.51 (1.38–1.65)	1.54 (1.41–1.68)	1.45 (1.32–1.59)
	130–159	1361	403	47.16	1.85 (1.65–2.09)	1.88 (1.67–2.11)	1.71 (1.52–1.94
	≥160	618	216	58.57	2.29 (1.98–2.66)	2.33 (2.01–2.70)	2.12 (1.82–2.47)
**Stroke**							
	<55	5203	280	7.31	1 (ref.)	1 (ref.)	1 (ref.)
	55–69	5894	351	8.02	1.08 (0.92–1.26)	1.09 (0.93–1.28)	1.09 (0.93–1.27)
	70–99	9696	574	7.95	1.08 (0.93–1.24)	1.09 (0.95–1.26)	1.07 (0.93–1.24)
	100–129	4150	289	9.36	1.25 (1.06–1.47)	1.26 (1.07–1.49)	1.17 (0.99–1.39)
	130–159	1361	119	12.01	1.57 (1.27–1.95)	1.60 (1.29–1.98)	1.43 (1.14–1.78)
	≥160	618	61	13.88	1.89 (1.43–2.49)	1.88 (1.43–2.49)	1.68 (1.26–2.23)
**Heart failure**							
	<55	5203	800	21.48	1 (ref.)	1 (ref.)	1 (ref.)
	55–69	5894	979	22.97	1.04 (0.95–1.15)	1.06 (0.97–1.16)	1.06 (0.96–1.16)
	70–99	9696	1528	21.73	0.98 (0.90–1.07)	1.00 (0.92–1.09)	0.99 (0.91–1.08)
	100–129	4150	747	25.00	1.12 (1.01–1.23)	1.13 (1.03–1.25)	1.09 (0.98–1.20)
	130–159	1361	292	30.24	1.32 (1.15–1.51)	1.34 (1.17–1.54)	1.26 (1.09–1.44)
	≥160	618	158	37.79	1.72 (1.45–2.04)	1.71 (1.44–2.03)	1.60 (1.34–1.91)
**Cardiovascular death**							
	<55	5203	150	3.84	1 (ref.)	1 (ref.)	1 (ref.)
	55–69	5894	181	4.04	1.04 (0.84–1.29)	1.05 (0.85–1.31)	1.05 (0.84–1.30)
	70–99	9696	353	4.78	1.25 (1.03–1.51)	1.27 (1.05–1.54)	1.22 (1.01–1.49)
	100–129	4150	180	5.67	1.46 (1.17–1.81)	1.48 (1.19–1.84)	1.31 (1.04–1.64)
	130–159	1361	65	6.31	1.58 (1.18–2.12)	1.62 (1.21–2.18)	1.35 (0.99–1.83)
	≥160	618	36	7.82	2.06 (1.43–2.97)	2.06 (1.43–2.97)	1.71 (1.17–2.48)
**All-cause mortality**							
	<55	5203	672	17.21	1 (ref.)	1 (ref.)	1 (ref.)
	55–69	5894	695	15.50	0.90 (0.81–1.00)	0.91 (0.82–1.01)	0.91 (0.81–1.01)
	70–99	9696	1307	17.69	1.05 (0.95–1.15)	1.07 (0.97–1.17)	1.04 (0.95–1.15)
	100–129	4150	641	20.20	1.17 (1.05–1.30)	1.19 (1.07–1.33)	1.09 (0.98–1.22)
	130–159	1361	227	22.02	1.25 (1.08–1.46)	1.28 (1.10–1.49)	1.13 (0.97–1.32)
	≥160	618	119	25.85	1.56 (1.29–1.90)	1.56 (1.28–1.89)	1.38 (1.13–1.68)

^a^Adjusted for age, sex, BMI, smoking, alcohol drinking, exercise, income status, hypertension, and estimated glomerular filtration rate.

^b^Adjusted for age, sex, BMI, smoking, alcohol drinking, exercise, income status, hypertension, estimated glomerular filtration rate, fasting glucose levels, use of insulin, and duration of diabetes.

^c^Adjusted for age, sex, BMI, smoking, alcohol drinking, exercise, income status, hypertension, estimated glomerular filtration rate, fasting glucose levels, use of insulin, duration of diabetes, and use of statin.

CI, confidence interval; HR, hazard ratio; IR, incidence ratio.

### Risk of CVE according to LDL-C and age category

Table 3 shows HRs of re-PCI, stroke, HF, cardiovascular death, and all-cause mortality according to LDL-C level and age category in the adjusted model. Using LDL-C level < 55 mg/dL as reference, HRs for re-PCI and stroke increased linearly according to LDL-C level in the population under age 65. However, in the population aged ≥ 65 years, results were different. IR and HR for re-PCI and stroke were the lowest in those with LDL-C level 55–69 mg/dL. HRs for re-PCI and stroke in those with LDL-C level 55–69 mg/dL were 0.97 (95% CI: 0.85–1.11) and 0.96 (95% CI: 0.79–2.23), respectively, with LDL-C < 55 mg/dL as the reference. Trends in the incidence of HF, cardiovascular death, and all-cause mortality were similar and unrelated to the age groups.

**Table 3:** Risk of re-PCI, stroke, heart failure, cardiovascular death, and all-cause of mortality in patients with type 2 diabetes mellitus according to low-density lipoprotein cholesterol (LDL-C) category and age.

		LDL-C	*N*	Event	IR	Model 3 HR (95% CI)^a^
**Re-PCI**	Age < 65					
		<55	2939	494	24.39	1 (ref.)
		55–69	3245	647	28.79	1.20 (1.07, 1.35)
		70–99	5403	1225	33.18	1.36 (1.23, 1.51)
		100–129	2267	602	39.70	1.57 (1.39, 1.77)
		130–159	741	246	51.95	1.97 (1.69, 2.30)
		≥160	353	136	64.44	2.42 (2.00, 2.94)
	Age ≥ 65					
		<55	2264	403	27.16	1 (ref.)
		55–69	2649	455	25.71	0.97 (0.85, 1.11)
		70–99	4293	909	32.48	1.21 (1.08, 1.37)
		100–129	1883	439	36.38	1.30 (1.14, 1.50)
		130–159	620	157	41.21	1.41 (1.17, 1.71)
		≥160	265	80	50.72	1.75 (1.37, 2.23)
**Stroke**	Age < 65					
		<55	2939	83	3.7175	1 (ref.)
		55–69	3245	130	5.2393	1.40 (1.07, 1.85)
		70–99	5403	204	4.9211	1.31 (1.02, 1.69)
		100–129	2267	105	6.0079	1.52 (1.14, 2.04)
		130–159	741	41	7.216	1.72 (1.18, 2.52)
		≥160	353	24	9.2976	2.25 (1.42, 3.57)
	Age ≥ 65					
		<55	2264	197	12.3339	1 (ref.)
		55–69	2649	221	11.6436	0.96 (0.79, 1.16)
		70–99	4293	370	12.0424	0.97 (0.81, 1.15)
		100–129	1883	184	13.7396	1.03 (0.84, 1.27)
		130–159	620	78	18.4596	1.30 (0.92, 1.71)
		≥160	265	37	20.3981	1.44 (1.01, 2.06)
**Heart failure**	Age < 65					
		<55	2939	358	16.5054	1 (ref.)
		55–69	3245	416	17.1374	1.02 (0.89, 1.18)
		70–99	5403	671	16.5722	0.98 (0.86, 1.12)
		100–129	2267	322	18.9874	1.08 (0.93, 1.26)
		130–159	741	119	21.5214	1.17 (0.95, 1.45)
		≥160	353	76	30.6538	1.74 (1.35, 2.23)
	Age ≥ 65					
		<55	2264	442	28.4229	1 (ref.)
		55–69	2649	563	30.6932	1.08 (0.96, 1.23)
		70–99	4293	857	28.7265	0.99 (0.88, 1.11)
		100–129	1883	425	32.8882	1.09 (0.95, 1.25)
		130–159	620	173	41.9074	1.32 (1.10, 1.58)
		≥160	265	82	48.1888	1.50 (1.18, 1.90)
**Cardiovascular death**	Age < 65					
		<55	2939	46	2.0398	1 (ref.)
		55–69	3245	55	2.1802	1.05 (0.71, 1.56)
		70–99	5403	96	2.2802	1.10 (0.77, 1.56)
		100–129	2267	41	2.3031	1.03 (0.67, 1.57)
		130–159	741	21	3.6082	1.46 (0.87, 2.46)
		≥160	353	15	5.6034	2.34 (1.29, 4.21)
	Age ≥ 65					
		<55	2264	104	6.3083	1 (ref.)
		55–69	2649	126	6.4245	1.04 (0.81, 1.35)
		70–99	4293	257	8.0915	1.28 (1.02, 1.61)
		100–129	1883	139	9.9775	1.43 (1.10, 1.86)
		130–159	620	44	9.8054	1.30 (0.91, 1.88)
		≥160	265	21	10.9012	1.44 (0.89, 2.32)
**All-cause mortality**	Age < 65					
		<55	2939	196	8.6915	1 (ref.)
		55–69	3245	180	7.1353	0.81 (0.67, 1.00)
		70–99	5403	346	8.2182	0.95 (0.80, 1.13)
		100–129	2267	157	8.819	0.97 (0.78, 1.20)
		130–159	741	61	10.4808	1.05 (0.79, 1.41)
		≥160	353	39	14.569	1.53 (1.08, 2.16)
	Age ≥ 65					
		<55	2264	476	28.8728	1 (ref.)
		55–69	2649	515	26.2591	0.94 (0.83, 1.07)
		100–129	1883	961	30.2565	1.08 (0.97, 1.21)
		130–159	620	484	34.7418	1.15 (1.01, 1.31)
		≥160	265	166	36.9932	1.17 (0.97, 1.40)

^a^Adjusted for age, sex, BMI, smoking, alcohol drinking, exercise, income status, hypertension, estimated glomerular filtration rate, fasting glucose levels, use of insulin, duration of diabetes, and use of statin.

CI, confidence interval; HR, hazard ratio; IR, incidence ratio.

## Discussion

Our study highlights the inverse association between LDL-C level and CVE risk for secondary prevention in T2DM patients with preexisting CVD. Current guidelines recommend an LDL-C goal ≤ 55 mg/dL for patients with very high-risk ASCVD regardless of the patient’s age (10, 11, 12, 13). However, our data revealed that the optimal LDL-C level for preventing CVE was 55–70 mg/dL, not under 55 mg/dL, in the population aged above 65 years. Therefore, the idea of lowering LDL-C as much as possible in elderly patients should be reconsidered.

In the population under age 65, outcomes of re-PCI, stroke, and cardiovascular death were inversely correlated with LDL-C levels in a linear manner. As postulated, the risk of CVE was the lowest in the population with LDL less than 55 mg/dL. Similar to outcomes of FOURIER (6), ODYSSEY OUTCOMES (7), and SPIRE (16) clinical trials, the cardiovascular benefit of low LDL-C levels was reproduced in our study using real-world data. Likewise, a previous systematic review has revealed that each millimole per liter (40 mg/dL) decrease in LDL-C level is associated with a 4.6% lower 5-year major coronary event rate for secondary prevention (5). Plus, a meta-analysis has evaluated a total of 18,686 patients with diabetes (1466 type 1 and 17,220 type 2) for a mean of 4.3 years, and found that there is a significant 9% reduction in all-cause mortality and a 21% reduction in coronary death or myocardial infarction, coronary revascularization, and stroke for each mmol per liter (40 mg/dL) of LDL-C reduction (17).

However, the optimal range of LDL-C varied by age group in our study. For the prevention of re-PCI and stroke in the elderly, the optimal LDL-C range was 55–69 mg/dL, not less than 55 mg/dL. ‘The lower, the better’ was applicable only to the population under 65 years old. It is well known that lipid lowering is as effective in reducing CVE in elderly patients as young patients for secondary prevention (18, 19, 20, 21). However, more studies should be performed to determine the optimal LDL-C level for bringing an additional benefit to the elderly patients.

A sub-analysis of the IMPROVE-IT trial with patients over 75 years old showed a reduction in CVE in the group with a mean LDL-C of 55.1 mg/dL compared to the one with a mean LDL of 68.7 mg/dL (19). These findings are in contrast with our results. The difference between IMPROVE-IT (19) and ours might have been caused to the difference in ethnicity. A meta-analysis report suggests that a target LDL-C level of less than 69 mg/dL is needed to reduce coronary atherosclerotic plaque in western populations, while a level of less than 84 mg/dL may be sufficient for Asians (22). Furthermore, there are differences in plaque morphology between East Asian and White patients (23). Our data indicate that a strict LDL-C lowering may not be as effective in elderly Asians as it is in White patients. To support these findings, a prospective prevention trial needs to be conducted.

Regarding the outcome for HF, ‘the lower, the better’ was not valid either. The optimal LDL-C level was 70–99 mg/dL in all age groups for preventing HF. Several previous studies have suggested that low total cholesterol levels are associated with increased mortality in patients with HF (24, 25, 26). In particular, Charach *et al.* (24) showed that low initial LDL-C level was a significant predictor of worse outcomes of both ischemic and nonischemic chronic HF in an elderly chronic HF cohort. Furthermore, a Korean adult study has demonstrated a U-curve association between LDL-C and HF mortality, with an optimal range of LDL-C at 130–159 mg/dL (25). Strict lowering of LDL-C might not be effective for the prevention or prognosis of HF, even for patients with established ASCVD. Several explanations can be offered for these findings. One explanation is that it might have a relation with the use of statins for CVD prevention. Coenzyme Q, an essential product of cardiac mitochondrial respiration, is reduced in congestive HF. Statin treatment can reduce coenzyme Q levels and it might be potentially harmful to patients with HF (27). Plus, lipoproteins are natural nonspecific buffers of endotoxin. Their binding to endotoxin can reduce lipopolysaccharide bioactivity and diminish immune activation known to impair chronic HF (26).

Regarding all-cause mortality, the optimal LDL-C level was 55–69 mg/dL in this very high-risk population. Several population-based studies have already shown a non-linear association between all-cause mortality and LDL-C levels (28, 29). A study on Koreans who were not taking statins has demonstrated a U-curve association between LDL-C and all-cause mortality, with an optimal LDL-C range at 140–159 mg/dL (25). Likewise, the Kangbuk Samsung Health Study on 347,971 individuals who were not taking statins highlighted that the group with an optimal LDL-C (130–159 mg/dL) had the lowest all-cause mortality (30). While the exact mechanism remains to be elucidated, several possibilities could explain this finding. It has been suggested that frailty, illnesses, and malnutrition are associated with lower cholesterol levels (31, 32). Higher LDL-C levels might reflect better nutritional and health status, which are likely related to better tolerance of acute medical stress (33, 34). Protective effects of LDL-C against cancer and infection have also been revealed (35, 36, 37). Malnutrition and frailty issues have more adverse impacts on the elderly. Therefore, we speculate that non-linear associations of LDL-C with all-cause mortality in this study might be due to the aforementioned cholesterol paradox.

The strength of this study is the use of the mass population. The NHIS database represents the entire Korean population. We analyzed nearly 27,000 people with T2DM with a history of prior PCI. With the aid of such big data, the present recommendation guideline for secondary prevention of lowering LDL-C to target has been proven valuable in the real-world setting for the population less than 65 years old. As proven in our data, an optimal LDL-C is crucial for survival benefits and quality of life.

This study has some limitations. First, the Korean NHIS database does not provide the assessment of LDL-C using blood samples. Therefore**,** LDL-C was calculated using the Friedewald formula. This calculation is only valid when the concentration of triglycerides is less than 400 mg/dL. It is not precise when LDL-C is very low (<50 mg/dL) (13). Although subjects with triglyceride greater than or equal to 400 mg/dL were excluded, those with very low LDL-C were included. In other words, although a lot of studies using the Korean NHIS database have published studies based on LDL-C using Friedewald formula, there might be an inconsistency between the calculated LDL-C and LDL-C assessed by blood samples.

Second, this study presumed that clinical factors used in this study were unchanged until the occurrence of the CVE or death. However, due to the nature of data sources, the LDL-C level was investigated in a single health screening examination. It is quite questionable that patients could sustain the observed cholesterol level and other indexes until the endpoint of this study. To conclusively demonstrate the benefit of optimal LDL-C levels in preventing further CVE, future studies are needed to show how reducing LDL-C levels to the optimal ranges compared to controls reduces its occurrence.

Third, this study was conducted only in South Korea. Results might not be applicable to other populations. Lastly, patients with severe disabilities might have difficulties undergoing health screening examinations. Therefore, they might not be included in the analysis.

## Conclusion

T2DM patients with established ASCVD are at particularly high risk for recurrent CVE. LDL-C level less than 55 mg/dL was optimal for secondary prevention of CVE in the population aged less than 65 years old. However, the optimal level for preventing recurrent PCI and stroke was 55–69 mg/dL in patients over 65 years old.

## Supplementary Materials

Supplementary Tables

Supplementary Figure

## Declaration of interest

Not applicable.

## Funding

This research received no specific grant from any funding agency in the public, commercial, or not-for-profit sectors

## Availability of data and materials

The data that support the findings of this study are available in Korean National Health Insurance Data Sharing Service at https://nhiss.nhis.or.kr/bd/ab/bdaba000eng.do.

## Author contribution statement

Kyungdo Han and Hyuk-Sang Kwon contributed to the study concept and design, data extraction and assessment, data synthesis, statistical analysis, and interpretation of data. Chaiho Jeong contributed to study concept and design and writing, and interpretation of data. Bongseong Kim contributed to data extraction and assessment, and data synthesis. Jinyoung Kim, Hansang Baek, Mee Kyoung Kim, Tae-Seo Sohn, Ki-Hyun Baek, Ki-Ho Son, and Hyun-Shik Son contributed to study concept and design. All authors reviewed and edited the manuscript. Kyungdo Han and Hyuk-Sang Kwon are the guarantors of this work and, as such, had full access to all the data in the study and take responsibility for the integrity of the data and the accuracy of the data analysis.
