# Supplementary material for: Optimal LDL cholesterol levels in young and old patients with type 2 diabetes for secondary prevention of cardiovascular diseases are different
Source: Endocr Connect. 2023 Sep 27;12(11):e230142. doi: 10.1530/EC-23-0142 (PMC10563641; doi:10.1530/EC-23-0142)
Supplement: Supplementary Tables [file supplementary_tables.pdf]

Supplementary table 1

Definitions of comorbidities and outcomes

Outcome	Definition
Diabetes mellitus	Admission ≥1 or outpatient clinic ≥1 with ICD-10 code E11-E14 and prescription of anti-diabetic drug (metformin, sulfonylureas, meglitinides, dipeptidyl peptidase 4 inhibitors, thiazolidinediones, alpha-glucosidase inhibitors, and insulin)
PCI	Procedure codes corresponding to M6551–M6554, M6561–M6564, M6571, and M6572
Stroke	Admission ≥1 with ICD-10 code I63 or I64
HF	Admission ≥1 with ICD-10 code I50
All-cause mortality	Death record provided by the Statistics Korea
CV-death	Death record provided by Statistics Korea whose death reason was I00-I99 (Diseases of the circulatory system)

Supplementary table 2

Baseline characteristics of subjects according to the low-density lipoprotein cholesterol (LDL-C) levels in male

	LDL-C < 55 (N=4,090)	LDL-C < 70 (N=4,386)	LDL-C 70–99 (N=6,885)	LDL-C 100–129 (N=2,791)	LDL-C 130–159 (N=889)	LDL-C ≥ 160 (N=384)	p-value
Baseline LDL-C mg/dL)	42.8 ± 9.8	62.2 ± 4.3	82.7 ± 8.4	112.0 ± 8.5	141.7 ± 8.5	210.5 ± 12.5	<.0001
Age (years)	61.7 ± 9.0	61.8 ± 8.8	61.5 ± 9.0	61.6 ± 9.2	61.2 ± 9.2	59.8 ± 9.9	0.001
Body mass index (kg/m2)	25.0 ± 2.8	25.1 ± 2.9	25.2 ± 2.9	25.2 ± 2.9	25.1 ± 2.9	25.2 ± 3.1	0.028
Fasting glucose (mg/dL)	134.0 ± 42.5	134.1 ± 41.9	135.8 ± 42.4	139.9 ± 47.5	144.7 ± 48.8	153.1 ± 59.3	<.0001
eGFR (mL/min/1.73 m2)	81.3 ± 40.9	79.9 ± 33.1	80.6 ± 33.6	80.6 ± 46.5	80.4 ± 46.8	80.6 ± 27.6	0.717
Baseline TC (mg/dL)	118.6 ± 17.6	134.4 ± 17.0	155.6 ± 18.0	187.6 ± 18.3	218.7 ± 19.6	262.6 ± 53.3	<.0001
Baseline HDL-C (mg/dL)	45.8 ± 15.0	45.6 ± 12.9	46.5 ± 15.4	46.7 ± 11.0	47.4 ± 32.0	51.6 ± 38.6	<.0001
Baseline TG (mg/dL)	129.9 (127.8 - 132.1)	118.0 (116.2 - 119.7)	119.7 (118.4 - 121.1)	131.0 (128.7 - 133.2)	141.9 (137.7 - 146.2)	157.2 (150.5 - 164.2)	<.0001
Hypertension	3,381 (82.7)	3,633 (82.8)	5,708 (82.9)	2,249 (80.6)	730 (82.1)	304 (79.2)	0.048
Current smoker	1,008 (24.7)	986 (22.5)	1,593 (23.1)	705 (25.3)	282 (31.7)	129 (33.6)	<.0001
Alcohol drinking	1,737 (42.5)	1,808 (41.2)	2,726 (39.6)	1,123 (40.2)	349 (39.3)	152 (39.6)	0.012
Regular exercise	1,121 (27.4)	1,224 (27.9)	1,872 (27.2)	724 (25.9)	221 (24.9)	91 (23.7)	0.153
Income (lower 25%)	759 (18.6)	826 (18.8)	1,299 (18.9)	566 (20.3)	193 (21.7)	110 (28.7)	<.0001
On insulin treatment	925 (22.6)	883 (20.1)	1,373 (19.9)	526 (18.9)	182 (20.5)	85 (22.1)	0.003
On statin treatment	3,892 (95.2)	4,145 (94.5)	6,098 (88.57)	1,938 (69.4)	513 (57.7)	235 (61.2)	<.0001
Duration of diabetes (years)	4.9 ± 3.4	4.6 ± 3.4	4.5 ± 3.5	4.4 ± 3.4	4.3 ± 3.4	3.8 ± 3.4	<.0001

Data are expressed as the mean ± SD, median (25–75%), or n (%) eGFR, estimated glomerular filtration rate; HDL, high-density lipoprotein; LDL, low-density lipoprotein; TC, total cholesterol; TG, triglyceride

Supplementary table 3

Baseline characteristics of subjects according to the low-density lipoprotein cholesterol (LDL-C) levels in female

	LDL-C < 55 (N=1,113)	LDL-C < 70 (N=1,508)	LDL-C 70–99 (N=2,881)	LDL-C 100–129 (N=1,359)	LDL-C 130–159 (N=472)	LDL-C ≥ 160 (N=234)	p-value
Baseline LDL-C mg/dL)	43.7 ± 9.0	62.5 ± 4.3	83.3 ± 8.4	111.9 ± 8.3	142.4 ± 8.3	200.4 ± 94.1	<.0001
Age (years)	66.3 ± 7.8	66.8 ± 7.9	66.5 ± 7.9	66.3 ± 8.3	67.2 ± 8.2	66.1 ± 8.4	0.167
Body mass index (kg/m2)	24.9 ± 3.2	25.1 ± 3.3	25.1 ± 3.3	25.4 ± 3.4	25.5 ± 3.6	25.3 ± 3.5	0.001
Fasting glucose (mg/dL)	132.8 ± 44.1	131.4 ± 44.8	132.9 ± 44.0	136.7 ± 48.4	138.3 ± 49.9	148.4 ± 56.8	<.0001
eGFR (mL/min/1.73 m2)	75.6 ± 26.1	76.0 ± 24.1	74.7 ± 25.3	73.8 ± 23.5	71.8 ± 24.1	72.4 ± 25.1	0.006
Baseline TC (mg/dL)	122.7 ± 18.3	137.9 ± 16.1	160.1 ± 17.5	191.3 ± 17.4	224.7 ± 18.9	272.7 ± 37.1	<.0001
Baseline HDL-C (mg/dL)	49.4 ± 17.0	49.0 ± 15.4	49.6 ± 11.5	51.4 ± 25.0	51.0 ± 12.5	58.2 ± 55.3	<.0001
Baseline TG (mg/dL)	130.7 (126.9 - 134.7)	118.8 (116.0 - 121.7)	122.7 (120.7 - 124.8)	131.1 (128.1 - 134.2)	144.1 (138.8 - 149.6)	149.7 (141.6 - 158.3)	<.0001
Hypertension	952 (85.5)	1,294 (85.8)	2,442 (86.9)	1,170 (86.1)	395 (83.7)	198 (84.6)	0.479
Current smoker	30 (2.7)	47 (3.1)	97 (3.5)	47 (3.5)	20 (4.2)	11 (4.7)	0.641
Alcohol drinking	46 (4.1)	73 (4.8)	156 (5.6)	68 (5.0)	27 (5.72)	10 (4.27)	0.618
Regular exercise	196 (17.6)	280 (18.6)	506 (18.0)	216 (15.9)	62 (13.1)	31 (13.3)	0.023
Income (lower 25%)	227 (20.4)	296 (19.6)	533 (19.0)	253 (18.6)	85 (18.0)	40 (17.1)	0.748
On insulin treatment	335 (30.1)	433 (28.7)	753 (26.8)	346 (25.5)	121 (25.6)	60 (25.6)	0.088
On statin treatment	1,079 (97.0)	1,433 (95.0)	2,552 (90.8)	1,084 (79.8)	286 (60.6)	142 (60.7)	<.0001
Duration of diabetes (years)	6.0 ± 3.3	5.6 ± 3.4	5.3 ± 3.4	5.2 ± 3.4	5.3 ± 3.3	4.8 ± 3.3	<.0001

Data are expressed as the mean ± SD, median (25–75%), or n (%)
eGFR, estimated glomerular filtration rate; HDL, high-density lipoprotein; LDL, low-density lipoprotein; TC, total cholesterol; TG, triglyceride

Supplementary table 4

Risk of re-PCI, stroke, heart failure, CV death and all-cause of mortality in patients with type 2 diabetes mellitus according to low-density lipoprotein cholesterol (LDL-C) category and gender

		LDL-C	N	Event	IR	Model 3<br>HR (95% CI)***
**Re-PCI**	Male					
		<55	4090	713	25.97	1 (ref.)
		55-69	4386	862	29.20	1.14 (1.03, 1.26)
		70-99	6885	1572	34.39	1.31 (1.20, 1.44)
		100-129	2791	728	40.22	1.46 (1.31, 1.62)
		130-159	889	275	49.88	1.74 (1.51, 2.00)
		≥160	384	137	61.38	2.13 (1.77, 2.56)
	Female					
		<55	1113	184	24.09	1 (ref.)
		55-69	1508	240	22.54	0.95 (0.79, 1.15)
		70-99	2811	562	29.27	1.22 (1.04, 1.45)
		100-129	1359	313	34.28	1.36 (1.13, 1.67)
		130-159	472	128	42.23	1.62 (1.29, 2.04)
		≥160	234	79	54.26	2.04 (1.57, 2.67)
**Stroke**	Male					
		<55	4090	217	7.23	1 (ref.)
		55-69	4386	237	7.28	1.01 (0.84, 1.21)
		70-99	6885	378	7.39	1.02 (0.87, 1.21)
		100-129	2791	188	9.12	1.17 (0.96, 1.43)
		130-159	889	68	10.41	1.31 (0.99, 1.73)
		≥160	384	37	13.60	1.78 (1.25, 2.54)
	Female					
		<55	1113	63	7.62	1 (ref.)
		55-69	1508	114	10.14	1.32 (0.97, 1.80)
		70-99	2811	196	9.33	1.22 (0.92, 1.62)
		100-129	1359	101	9.84	1.23 (0.90, 1.69)
		130-159	472	51	15.11	1.71 (1.18, 2.50)
		≥160	234	24	14.33	1.65 (1.03, 2.66)
**Heart failure**	Male					
		<55	4090	603	20.6248	1 (ref.)
		55-69	4386	685	21.5895	1.04 (0.93, 1.16)
		70-99	6885	1001	20.0253	0.96 (0.87, 1.06)
		100-129	2791	453	22.5588	1.04 (0.92, 1.18)
		130-159	889	170	26.7714	1.20 (1.01, 1.43)
		≥160	384	98	38.1815	1.79 (1.44, 2.23)
	Female					
		<55	1113	197	24.6126	1 (ref.)
		55-69	1508	294	27.0002	1.10 (0.92, 1.32)
		70-99	2811	527	25.9149	1.06 (0.90, 1.25)
		100-129	1359	294	29.9989	1.20 (1.00, 1.44)
		130-159	472	122	36.8862	1.37 (1.09, 1.72)
		≥160	234	60	37.1687	1.41 (1.05, 1.88)
**CV death**	Male					
		<55	4090	116	3.7914	1 (ref.)
		55-69	4386	132	3.97122	1.05 (0.82, 1.34)
		70-99	6885	242	4.62967	1.21 (0.97, 1.51)
		100-129	2791	119	5.62055	1.31 (1.00, 1.70)
		130-159	889	36	5.33296	1.19 (0.81, 1.74)
		≥160	384	24	8.43404	1.96 (1.26, 3.07)
	Female					
		<55	1113	34	4.02785	1 (ref.)
		55-69	1508	49	4.22418	1.05 (0.68, 1.62)
		70-99	2811	111	5.14075	1.26 (0.86, 1.85)
		100-129	1359	61	5.77571	1.32 (0.87, 2.01)
		130-159	472	29	8.15296	1.63 (0.99, 2.70)
		≥160	234	12	6.82711	1.37 (0.71, 2.66)
**All-cause mortality**	Male					
		<55	4090	528	17.2574	1 (ref.)
		55-69	4386	537	16.1557	0.94 (0.83, 1.06)
		70-99	6885	939	17.9639	1.05 (0.95, 1.17)
		100-129	2791	441	20.8291	1.10 (0.97, 1.26)
		130-159	889	137	20.2949	1.05 (0.87, 1.28)
		≥160	384	72	25.3021	1.39 (1.08, 1.79)
	Female					
		<55	1113	144	17.0591	1 (ref.)
		55-69	1508	158	13.6208	0.81 (0.64, 1.01)
		100-129	2811	368	17.0432	1.01 (0.83, 1.22)
		130-159	1359	200	18.9368	1.06 (0.85, 1.31)
		≥160	472	90	25.3023	1.25 (0.96, 1.64)

IR, incidence ratio; HR, hazard ratio; CI, confidence interval
*** Adjusted for age, sex, BMI, smoking, alcohol drinking, exercise, income status, hypertension, estimated glomerular

filtration rate, fasting glucose levels, use of insulin, duration of diabetes and use of statin
