# Supplementary material for: Optimal LDL cholesterol levels in young and old patients with type 2 diabetes for secondary prevention of cardiovascular diseases are different
Source: Endocr Connect. 2023 Sep 27;12(11):e230142. doi: 10.1530/EC-23-0142 (PMC10563641; doi:10.1530/EC-23-0142)
Supplement: Supplementary Figure [file supplementary_figure.pdf]

Optimal LDL cholesterol level differs in young and old for secondary prevention

Patients

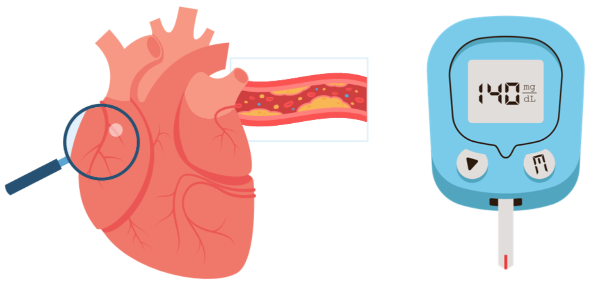

Type 2 diabetes patients
with established cardiovascular disease (CVD)

Method

Korean National Health Insurance System database

Results

Optimal range of LDL-C level

	Re-PCI	stoke	CV death	HF	All-cause death
Age < 65	<55	<55	<55	70-99	55-69
Age ≥ 65	55-69	55-69	<55	70-99	55-69

Aim

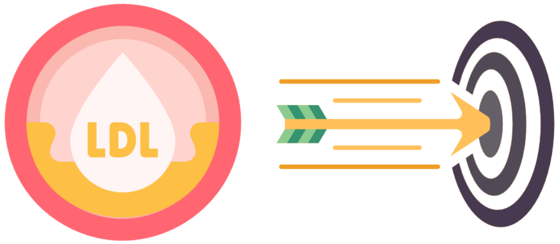

Optimal target of LDL-cholesterol

Outcome

New onset CVD

- **Event of recurrent PCI**
- **Newly diagnosed stroke or HF**
- **CV death**
- **All-cause death**

Conclusion

LDL-C level < 55 mg/dL was beneficial for preventing CVD in T2DM patients with established CVD aged < 65 years. However, the optimal level for preventing recurrent PCI and stroke was 55-69 mg/dL in patients over 65 years old.
